# The obstetric hematology joint clinic in Qatar: experience since 2020

**DOI:** 10.1177/26334941261435054

**Published:** 2026-04-11

**Authors:** Sarah A. Elkourashy, Naela Elmallahi, Lawal Bappa, Nurhan Elshafey, Sharon Jacob, Sara Osman Musa, Annamma Mathew, Salwa Abuyaqoub

**Affiliations:** Department of Hematology and Bone Marrow Transplant, National Centre for Cancer Care and Research (NCCCR), Hamad Medical Corporation (HMC), Doha 3050, Qatar; Weill Cornell Medicine – Qatar, Doha, Qatar; Department of Obstetrics and Gynecology, Women’s Wellness and Research Center (WWRC), HMC, Doha, Qatar; Department of Obstetrics and Gynecology, Women’s Wellness and Research Center (WWRC), HMC, Doha, Qatar; Department of Clinical Pharmacy and Pharmacology, HMC, Doha, Qatar; Division of Transfusion Medicine, Department of Laboratory Medicine and Pathology, HMC, Doha, Qatar; Department of Medical Genetics, HMC, Doha, Qatar; Department of Nursing, WWRC, HMC, Doha, Qatar; Department of Obstetrics and Gynecology, Women’s Wellness and Research Center (WWRC), HMC, Doha, Qatar

**Keywords:** hematologic disorders in pregnancy, high-risk pregnancy, integrated care model, multidisciplinary clinic, obstetric hematology, service evaluation, specialist antenatal care

## Abstract

**Background:**

Obstetric hematology subspeciality represents unique discipline that combines maternal–fetal medicine and hematology expertise to optimize care for pregnancy-associated and pre-existing hematologic disorders.

**Objective:**

To describe the implementation and clinical experience of the Obstetric Hematology Joint Clinic at the Women’s Wellness and Research Center (WWRC), a specialized tertiary women’s hospital in Qatar, since August 2020.

**Design and Methods:**

Single-center descriptive service evaluation using routinely collected data from August 2020 to December 2025, including patient encounters, diagnostic categories, and interventions delivered by the multidisciplinary team.

**Results:**

A total of 2,564 patient encounters were recorded over the study period, with steady annual growth. The clinic managed hemoglobinopathies, bleeding and thrombotic disorders, and pregnancy-related anemia and thrombocytopenia. Key interventions included intravenous iron therapy, anticoagulation management, transfusion support, and individualized multidisciplinary birth planning.

**Conclusion:**

The integrated clinic model improves coordinated care, enhances peripartum preparedness, and supports safe management of complex hematologic conditions during pregnancy and postpartum.

## Introduction

The field of obstetric hematology has emerged as a fundamental subspecialty in maternal healthcare, addressing the complex interplay between pregnancy and hematological disorders, where delays in recognition and fragmented care may worsen maternal–fetal outcomes. Pregnancy induces significant physiological changes in the hematologic system, which can exacerbate existing conditions or lead to new complications such as anemia, thrombocytopenia, or thromboembolic events.^1,2^ These complications, if not properly managed, can result in severe consequences for both the mother and the fetus, including increased risk of maternal mortality, preterm delivery, and fetal growth restrictions.^3,4^

The development of obstetric hematology as a discipline is crucial for improving maternal and fetal outcomes in high-risk pregnancies.^5^ It enables healthcare providers to offer specialized care tailored to the unique needs of pregnant women with blood disorders, thus reducing the risk of complications.^5^ Establishing the Obstetric Hematology Joint Clinic in Qatar significantly advances this field. Such a clinic provides a multidisciplinary approach to care, combining the expertise of hematologists, obstetricians, and maternal–fetal medicine specialists to ensure comprehensive and effective evidence-based management of these complex cases.

The importance of this subspecialty is underscored by the high rates of pregnancy-related hematological complications globally.^6^ For instance, conditions like pulmonary embolism remain a leading cause of maternal death in developed countries, while hemorrhage continues to dominate in developing regions. Thrombosis and thromboembolism were the leading causes of maternal death in the recent United Kingdom maternal mortality review.^7^ FIGO (International Federation of Gynecology and Obstetrics) reported that in 2020, nearly 800 women died daily from preventable pregnancy- and childbirth-related causes, with a global maternal mortality rate of 223 per 100,000 live births.^8^ These statistics highlight the critical need for specialized obstetric hematology services to enhance maternal and fetal outcomes worldwide.

The initiative for this service was inaugurated in 2019. After thorough planning, logistical preparations, and arrangements, the clinic was successfully launched in August 2020, with services available on a weekly basis every Tuesday from 8:00 AM to 3:00 PM. It operates within the outpatient department at the Women’s Wellness and Research Center (WWRC), a member of Hamad Medical Corporation (HMC), the principal public healthcare provider in Qatar. It serves pregnant women aged 18 years and above, providing acute and follow-up care. The clinic’s mission is to offer high-quality, up-to-date, evidence-based healthcare in Qatar to women with blood disorders during pregnancy and the early postnatal period through a multidisciplinary approach.

## Objectives

To provide an overview of the establishment, implementation, and operational experience of the Obstetric Hematology Joint Clinic at the Women’s Wellness and Research Center (WWRC) in Qatar, since its inception in August 2020.

## Scope of the clinic

The clinic’s scope of care encompasses many conditions. Firstly, pre-existing hematologic disorders including (a) hemoglobinopathies such as sickle cell anemia and thalassemia, (b) bleeding disorders as Von Willebrand disease (VWD), hemophilia carriers (A or B), hypo or dysfibrinogenemia and other rare coagulation factor deficiency, (c) thrombotic disorders due to either autoimmune causes or hereditary thrombophilia (e.g., Factor V Leiden mutation, Prothrombin G20210A mutation, protein C or S deficiency, antithrombin III deficiency), (d) bone marrow diseases including myeloproliferative neoplasms (MPNs), aplastic anemia, sideroblastic anemia, and survivors of hematological malignancies.

Secondly, pregnancy-related hematologic disorders that include (a) pregnancy-associated anemia such as iron deficiency anemia and megaloblastic anemia (folate or B12 deficiency), (b) pregnancy-associated thrombocytopenia, for example, gestational thrombocytopenia, immune thrombocytopenia (ITP) or as a part of critical conditions that need urgent admission as thrombotic thrombocytopenic purpura (TTP) or HELLP syndrome (hemolysis, elevated liver enzymes, low platelets), and (c) venous thromboembolism (VTE): Pregnancy is a hypercoagulable state, increasing the risk of deep vein thrombosis (DVT) or pulmonary embolism (PE).

## Methods

### Study design and setting

This study is a single-center descriptive service evaluation of the Obstetric Hematology Joint Clinic at the Women’s Wellness and Research Center (WWRC), a tertiary referral women’s health center in Qatar. The clinic was established in 2020 to provide integrated multidisciplinary care for pregnant and postpartum women with complex hematologic conditions. The clinic operates through joint consultations involving obstetricians and hematologists, with coordinated diagnostic evaluation, treatment planning, and follow-up.

### Study population and eligibility criteria

Women were eligible for inclusion if they were pregnant (at any gestational age) or in the immediate 6-week postpartum period and were referred to the Obstetric Hematology Joint Clinic between August 2020 and December 2025 for evaluation or management of a hematologic condition complicating pregnancy or the postpartum period. Eligible referrals included women with moderate to severe anemia, hemoglobinopathies, inherited or acquired bleeding disorders, thrombocytopenia, thromboembolic disease, or other complex coagulation abnormalities requiring specialist hematology input. Patients were included if their clinical management necessitated joint assessment by obstetric and hematology specialists, either for diagnostic clarification, treatment planning, or longitudinal follow-up.

Women were excluded if they were not pregnant or postpartum at the time of referral, if the referral was for isolated laboratory abnormalities that did not require specialist hematology consultation, or if the encounter was administrative in nature without direct clinical assessment. Patients with insufficient clinical documentation to allow meaningful evaluation were also excluded.

### Outcomes and descriptive analysis

The primary outcomes of interest were the spectrum of hematologic conditions managed, patterns of referral, and descriptive characteristics of the clinic population. Secondary outcomes included clinical management approaches and coordination of care following clinic implementation. Data were analyzed descriptively and presented without comparative statistical analysis.

### Ethical considerations

Service evaluation was done using aggregate and de-identified data. Formal patient consent and Institutional Review Board (IRB) approval were not required for this descriptive report in accordance with the institutional policy of Hamad Medical Corporation (HMC-IRB).

## Results

Service activity: 2564 patient encounters from August 2020 to December 2025.

### Conditions managed

- Pre-existing hematologic disorders: hemoglobinopathies (sickle cell disease, thalassemia), bleeding disorders (e.g., VWD, hemophilia carriers, dys/hypofibrinogenemia), hereditary/acquired thrombophilia, bone marrow diseases (e.g., MPNs, aplastic anemia, sideroblastic anemia), survivors of hematologic malignancy.
-Pregnancy-related disorders: iron deficiency anemia and megaloblastic anemia, gestational thrombocytopenia/ITP, critical microangiopathies (e.g., TTP, HELLP), and venous thromboembolism (DVT/PE).

Interventions typically delivered included: Anticoagulation management and peripartum bridging. Iron therapy optimization (oral/IV) and anemia clinics. Transfusion support and factor concentrate; viscoelastic-guided hemostasis (ROTEM/TEG) where indicated. Genetic counseling and prenatal diagnostics coordination (CVS/amniocentesis) for hereditary disorders. Multidisciplinary birth-planning meetings (34–36 weeks) and emergency preparedness drills.

#### Key observations

**Figure 1.**
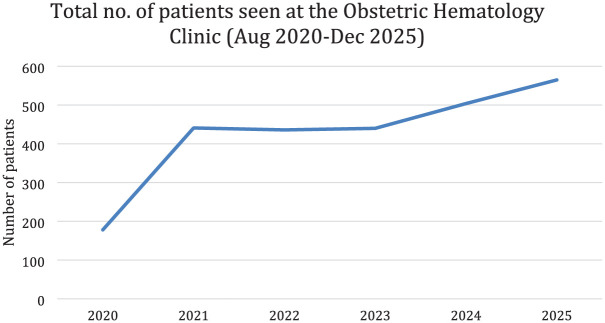
Number of patients seen at the Obstetrics Hematology Joint Clinic.

Consistent annual caseload with increasing service maturity since launch in August 2020 (Figure 1). Standardized multidisciplinary care pathways and earlier identification of alloimmunization/rare blood types through extended phenotyping and antigen matching. This led to improved coordination for high-risk deliveries via scheduled MDT plans and blood product mobilization.

### Clinic activity and patient characteristics

Between August 2020 and December 2025, a total of 2564 women were reviewed in the Obstetric Hematology Joint Clinic. The majority of patients were referred during early pregnancy, with referrals occurring across all trimesters, while a smaller proportion were seen in the postpartum period (6 weeks). Referrals originated primarily from antenatal outpatient clinics, followed by obstetric inpatient services and subspecialty obstetric teams.

Most patients required ongoing follow-up rather than a single consultation, reflecting the complexity of hematologic conditions managed.

### Spectrum of hematologic conditions

The most common indications for referral were moderate to severe anemia, followed by thrombocytopenia, hemoglobinopathies, and bleeding or coagulation disorders. A smaller proportion of referrals involved thromboembolic disease or complex coagulation abnormalities requiring individualized management. Several patients presented with more than one hematologic condition, necessitating coordinated care planning.

### Clinical management and multidisciplinary input

Management strategies included optimization of antenatal hematinic therapy, planning for peripartum transfusion support, coordination of anticoagulation management, and development of individualized delivery and postpartum care plans. Joint obstetric–hematology consultations facilitated timely decision-making regarding investigations, treatment escalation, and delivery planning, particularly for women with high-risk hematologic profiles.

## Discussion

Women presenting with these hematologic disorders during pregnancy face distinctive clinical challenges that demand vigilant antenatal monitoring and a carefully coordinated delivery plan, typically between 34 and 36 weeks of gestation. The multidisciplinary Obstetric Hematology Clinic integrates hematology, obstetrics, anesthesiology, transfusion medicine, clinical pharmacy, genetics, midwifery, and social workers to ensure safe, well-planned deliveries and to strengthen team communication and decision-making during emergencies. This specialized collaboration also drives the development of institutional guidelines and management protocols, promoting a comprehensive, patient-centered approach across the prenatal, intrapartum, and postpartum continuum of care. Since its inception, the clinic has managed plenty of diverse cases, reflecting the growing demand for such specialized services (Table 1). This consistent patient load underscores the clinic’s importance and effectiveness in addressing the needs of this vulnerable patient population (Figure 1).

**Table 1.** Annual activity of the Obstetric Hematology Joint Clinic (August 2020–December 2025).

Year	Patient encounters
2020 (August–December)	178
2021	441
2022	436
2023	440
2024	504
2025	565

Regarding the role of nursing in the clinic, the registered nurse conducts the initial nursing assessment, provides instructions on investigations, medications, and admission procedures if needed, educates patients on relevant topics, and ensures patient safety.

Clinical pharmacists’ role is integral to the obstetric hematology clinic, ensuring the safe and effective use of medications for pregnant women with hematologic conditions. They manage anticoagulation therapy, optimize iron supplementation, monitor drug interactions, assess teratogenicity risk, and educate patients on treatment adherence. In addition, they collaborate with medical teams to prevent and manage thromboembolic events, guide bridging therapy, ensure proper stocking of clotting factors, and contribute to emergency preparedness, particularly in cases such as postpartum hemorrhage. They educate and counsel lactating females on medication safety during breastfeeding.

The clinic collaborates with a genetic team consisting of geneticists and genetic counselors. Pregnant ladies affected or who have been carriers of hematological diseases such as alpha or beta thalassemia, sickle cell disease, hemophilia, VWD, dysfibrinogenemia, and other bleeding disorders are referred to the prenatal genetic clinic. They are provided with formal genetic counseling for the pregnant lady herself and her fetus’ status, counseling on prenatal genetic testing such as chorionic villus sampling and amniocentesis, as well as counseling on possible management options for an affected fetus. Care is coordinated between the obstetric, hematology, and neonatology teams for babies that may need special care at the time of labor or postnatal precautions.

Transfusion medicine plays a pivotal role in managing hematologic challenges during pregnancy. Routine antenatal blood typing and antibody screening in early and late gestation enables early detection of irregular antibodies and rare blood types, ensuring timely, individualized blood management. For alloimmunized patients with multiple or high-titer red cell antibodies, early fetal monitoring and preparation of antigen-negative blood are essential. To minimize the risk of alloimmunization, extended phenotyping, prophylactic antigen matching, and restrictive transfusion strategies are employed.

Qatar’s diverse population presents significant challenges for blood management, particularly in cases involving rare blood types and complex red cell antibodies, requiring multidisciplinary coordination for proper risk assessment, optimization of hematinic therapy, blood conservation, and early donor identification. Intraoperative cell salvage and autologous collection are planned when feasible while maintaining readiness for massive hemorrhage management through effective communication and contingency planning. Establishing clear protocols with the blood bank and having alternative plans for product shortages can help mitigate risks. Point-of-care viscoelastic testing of hemostasis: ROTEM (Rotational Thrombo-elastometry) and TEG (Thrombo-elastography) provide real-time guidance for transfusion decisions.^9^ Regular simulation drills and team training further enhance preparedness, coordination, and ensure the timely delivery of blood products and factor concentrates.

Beyond its clinical impact, the clinic has evolved into a dynamic educational hub, offering comprehensive interdisciplinary training for medical students, residents, and fellows. It serves as a core rotation in programs such as the Maternal Medicine and Critical Care Obstetric Fellowship. Trainees actively engage in case discussions, research, and conference presentations, fostering academic growth and clinical excellence.

By uniting patient-centered care, education, and research, the Obstetric Hematology Clinic has become a cornerstone of maternal healthcare in Qatar. Such a successful model not only advances local standards of practice but also provides a scalable framework for specialized obstetric hematology services worldwide.

This single-center experience suggests that a dedicated obstetric hematology service can systematize risk stratification, streamline access to specialized therapies (e.g., factor concentrates, IV iron), and enable coordinated intrapartum planning, including availability of antigen-matched blood and viscoelastic-guided transfusion algorithms. Implementation barriers included scheduling across services, product shortages, and coordination for rare phenotypes; these were mitigated through standardized MDT templates, early blood bank notification, and simulation drills. Future directions include prospective registry analyses of maternal and neonatal outcomes and benchmarking against international programs.

## Limitations

This study has several limitations. As a single-center, descriptive service evaluation, the findings may not be generalizable to other settings with different patient populations or healthcare structures. The absence of a comparator group and the retrospective nature of data collection preclude assessment of causal relationships or quantitative evaluation of clinical outcomes. In addition, the analysis focused on service delivery and care coordination rather than long-term maternal or neonatal outcomes. Future studies incorporating comparative designs, detailed figures with percentages of managed conditions, and standardized outcome measures would help further evaluate the impact of multidisciplinary obstetric hematology care.

## Conclusion

The Obstetric Hematology Joint Clinic represents a structured, multidisciplinary approach to the management of complex hematologic conditions during pregnancy and the postpartum period in a tertiary care setting. Our experience demonstrates the feasibility and practical value of coordinated obstetric–hematology care in supporting individualized management and continuity across the perinatal pathway. While derived from single-center experience, this model may inform the development of similar collaborative services in comparable healthcare settings, particularly where complex maternal hematologic conditions are frequently encountered.

## Declarations

**Ethics approval and consent to participate:** As per the Hamad Medical Corporation Institutional Review Board (HMC IRB), Service evaluations and Quality improvement projects do not require ethical oversight or consent to participate.

**Consent for publication:** Not applicable (no individual patient data/images).

**Availability of data and materials:** Not applicable.
